# Reliable and mobile all-fiber modular optical tweezers

**DOI:** 10.1038/s41598-020-77067-1

**Published:** 2020-11-18

**Authors:** Chaoyang Ti, Yao Shen, Minh-Tri Ho Thanh, Qi Wen, Yuxiang Liu

**Affiliations:** 1 Department of Mechanical Engineering, Worcester Polytechnic Institute, Worcester, MA 01609 USA; 2 Department of Physics, Worcester Polytechnic Institute, Worcester, MA 01609 USA

**Keywords:** Optical manipulation and tweezers, Fibre optics and optical communications, Optics and photonics

## Abstract

Miniaturization and integration of optical tweezers are attractive. Optical fiber-based trapping systems allow optical traps to be realized in miniature systems, but the optical traps in these systems lack reliability or mobility. Here, we present the all-fiber modular optical tweezers (AFMOTs), in which an optical trap can be reliably created and freely moved on a sample substrate. Two inclined optical fibers are permanently fixed to a common board, rendering a modular system where fiber alignments are maintained over months. The freely movable optical trap allows particles to be trapped in their native locations. As a demonstration, we applied AFMOTs to trap and deform freely floating individual cells. By the cell mechanical responses, we differentiated the nontumorigenic breast epithelial cell line (MCF10A) from its cancerous PTEN mutants (MCF10 PTEN-/-). To further expand the functionalities, three modalities of AFMOTs are demonstrated by changing the types of fibers for both the optical trap creation and particle position detection. As a miniature and modular system that creates a reliable and mobile optical trap, AFMOTs can find potential applications ranging from point-of-care diagnostics to education, as well as helping transition the optical trapping technology from the research lab to the field.

## Introduction

Optical tweezers (OTs) are important tools widely applied in biology^1–5^, material science^6^, and physics^7–10^ since the pioneering work by Ashkin and co-workers in early 1970s^8^. Miniaturization and integration have been considered as one of the most important trends for the development of OTs^11^. Miniature OTs with all the components integrated can be implemented in systems such as integrated analytical devices^12^ and, more importantly, can help transition the optical trapping technology from the research lab to practical applications. Despite the importance, miniaturization and integration of optical tweezers are challenging so far. The most commonly used OTs, referred to as traditional OTs, are built on a microscope platform with a strongly focused laser beam^8,13,14^ and an objective lens with a high numerical aperture (NA), the latter of which is required for both creating a trap and detecting the trapped particle position^15,16^. Therefore, traditional OTs inherit the limitations of the high NA objective and free-space optics, including the bulky size, short working distance, difficulty to be integrated, and susceptibility to environmental fluctuations.

Optical trapping systems based on optical fibers are one of the solutions to realizing miniature and integrated OTs. However, it is still challenging to maintain both the mobility and reliability of the optical trap in the fiber optical trapping systems. The most commonly used fiber OTs have a counter-propagating configuration, which is composed of two cleaved fibers aligned along a straight line either inside a groove^17,18^ or against a glass capillary^19–21^ on a substrate. Such a modular system can reliably create an optical trap between the fiber tips without fiber realignments. However, the optical trap cannot be freely moved and confined close to the substrate, limiting its capability to be integrated with other system components. The traps created by cleaved single fibers^22^ or fiber bundles^23,24^ can be moved around but cannot confine particles in three dimensions. To achieve freely movable three-dimensional (3D) traps, specialty fibers are commonly employed. Optical trapping has been realized by single-core fibers with tips patterned by focused ion beam lithography (FIB)^25,26^, tapered by heating-and-drawing technology^27,28^, or chemically etched^29,30^. 3D optical trapping has also been demonstrated using a multi-core fiber with a specialty tip made by fiber side grinding^31^ or the heat-and-draw method^32^, as well as using multiple specialty fibers whose end faces are shaped by FIB milling^33^ or two-photon lithography^34^. These specialty fibers have delicate structures, raising concerns of the lifetime and reliability of the trapping performance. The reliability of the fiber-based optical trap performance also influences the particle position detection capability. In all the abovementioned work, such detection is done by using an objective lens to image the trapped particles onto either a position-sensitive detector or a high-speed camera. The fiber drifts and the trapping efficiency fluctuations could bring challenges to the analysis of position detection signals. Although some efforts have been made to detect the trapped particle position with optical fibers, the particle displacements in transverse and axial directions were coupled in the fiber detection signals^33,35^, limiting the detection faithfulness. We reported a fiber-based, objective-lens-free detection scheme with a nm resolution in a non-modular system^36^. However, in practice, the fibers held by the translation stages drift over time, limiting the reliability of both the trap performance and fiber-based detection sensitivity. There is an urgent need for a modular fiber optical trapping system that creates a freely moveable optical trap, while such fiber optical tweezers have yet to be seen. A modular system enables reliable creation of optical trap with minimum influences from the environment fluctuations and system drift, while a freely move optical trap allows the particles to be trapped at desired locations. For example, such a modular system enables biological cells to be trapped and studied over a long period at their native locations in the cultured media or tissues.

Here, we present a maintenance-free, all-fiber modular optical tweezers (AFMOTs) system that can reliably create an optical trap that is freely movable on the sample substrate. It consists of two inclined optical fibers pre-aligned and permanently fixed to a centimeter-scale common board, and the optical trap is located at the tips of the two fibers, well below the common board. Such a modular design eliminates the need of the fiber alignment maintenance and free-space optics, while the mobility of the trap is reserved with the performance reliability. Compared with the counter-propagating fiber trapping systems, which is also a modular system, the optical trap created by AFMOTs can be freely moved inside the medium, pick up a particle lying on a substrate, and move the particle around on the substrate. In addition to the system integration with devices such as lab-on-a-chip devices and microscopes, the combination of trap mobility and reliability bestows on the AFMOTs great potential in finding applications that are challenging for other fiber optical tweezers. For example, AFMOTs can be used in the biomechanical investigation of cells in their original locations in the cultured media or tissues, as well as long-period applications such as the cell studies during its growth and division. As a demonstration for the applications, we used AFMOTs as a functional tool to “probe” the mechanical properties of cells in their original locations in the cultured medium. By applying AFMOTs to trap and deform individual cells, we differentiated the nontumorigenic breast epithelial cell line (MCF10A) from its cancerous PTEN mutants (MCF10 PTEN-/-) based on the measured cellular deformability, while the cell deformability can be used as a biomarker of cancer and many other types of diseases^21^. We estimated Young’s modulus of individual cells, which is in a good agreement with that published in literature. It is noted that, as a material characterization tool, AFMOTs interrogate the single-cell deformability on-site and free of labeling. Such a method is advantageous compared with the standard proteomic techniques requiring labeling reagents for detection^37^ and the physical-contact-based mechanical probing methods such as Atomic Force Microscope (AFM)^38^, both of which have been used for disease diagnosis. To further elaborate on the potential of AFMOTs, we have demonstrated various modalities of AFMOTs by using different types of fibers, with each modality suitable for different applications. The nm-resolution, MHz-bandwidth position detection based on optical fibers, which was demonstrated before with a non-modular system^36^, has also been confirmed with the AFMOTs. Exemplifying the miniature trend of OTs development, AFMOTs can potentially bring the optical trapping technology out of research labs to benefit the masses in point-of-care applications, disease diagnosis, and education.

## Results

### Design and working principles

**Figure 1:**
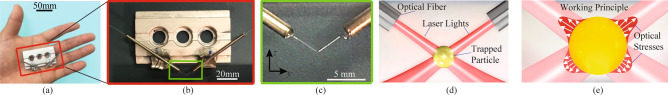
Prototype of AFMOTs within the palm of a hand (**a**) and its close-up views (**b**,**c**). The fibers are located in the *yz* plane. (**d**) Schematic of the optical beam configuration at the trap. (**e**) Working principles of a 3D trap and particle deformation. The white arrows and the red envelopes stand for the direction and the magnitude, respectively, of the radiation pressure (optical stress), applied on the particle surface. The fibers in the system photos and schematics are cleaved single-mode optical fibers at 980 nm.

The AFMOTs are modular optical trapping systems without any mechanically moving parts inside. The design is based on an inclined dual-fiber optical tweezers setup, the latter of which has been discussed in previous works^36,39–42^. Briefly, two optical fibers with an inclined angle (defined in the Method) are pre-aligned and permanently fixed on a common board (Fig. 1), serving as a system block. Thus, a 3D optical trap is created reliably and repeatably by a modular system with a footprint of 85 × 50 × 7 mm^3^, as shown in Fig. 1a–c. The modular form factor makes it straightforward to mount the AFMOTs on microscopes. The detailed fabrication process, including how to ensure and maintain the alignment of the two fibers, can be found in the supplementary information. The position of the optical trap can be easily maneuvered by controlling the position of the system block. In addition to creating a trap, the AFMOTs enable the fiber-based particle position detection with a resolution of 2 nm^36^. The new modular system design solves the most important limitations of our previous work, which are the fiber drift over time and the corresponding re-alignments. Combined with the simultaneous trapping and detection functions based on optical fibers, the AFMOTs not only have significantly improved robustness and reliability, but provide great potential for the optical trapping technology to be used outside a lab in a push-button system.

A 3D trap is created slightly lower than the beam axis intersection (Fig. 1d), with principles discussed in our previous work^36,41,42^. In addition to being trapped, elastic particles such as biological cells can be deformed in response to the optical forces, which are the surface integrals of radiation pressure (optical stress) applied by the AFMOTs. Arising from the transfer of momentum from the incident photons to the trapped particles, the optical stress at each point on the particle surface is along the outward-pointing surface normal, if the particle has a higher refractive index than the medium^21^. Thus, two inclined optical beams result in four optical stresses, as shown in Fig. 1e, resulting in the elongation of the particle along the *y* direction.

### Interrogation of cell deformability by AFMOTs

Cell mechanical properties, such as elastic properties, are related to cell malfunctions^43^. Therefore, how easy a cell can be deformed is determined by its mechanical properties and can serve as a biomarker of diseases^21^. Previously, traditional optical tweezers^44,45^ and counter-propagating fiber optical tweezers^21,46^ have been used to study deformability of cells, but they are limited by the free-space optics and the trap location close to alignment substrates, respectively. By comparison, with a modular form factor and a freely movable trap, AFMOTs are an ideal tool to interrogate cell deformability particularly for point-of-care and possibly in vivo applications. Here, we experimentally demonstrate the capability of AFMOTs to differentiate the cancerous MCF10A PTEN-/- human breast epithelial cell line (see Methods) from the MCF10A non-tumorigenic one.

**Figure 2:**
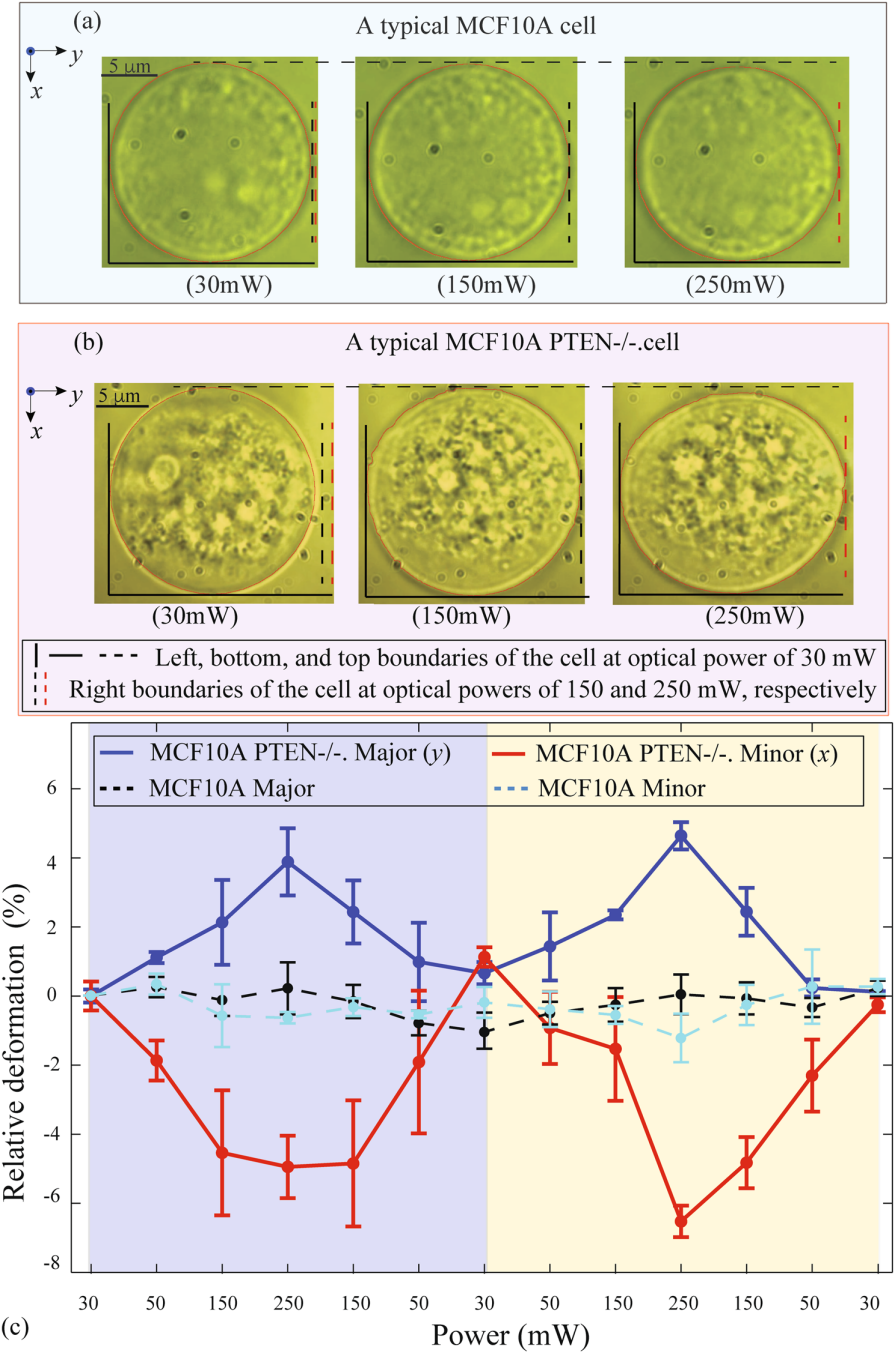
Interrogation of cell deformability by AFMOTs with cleaved fibers. The typical microscopic images of a deformed MCF10A (**a**) and MCF10A PTEN-/- cell (**b**) under the optical power of 30, 150, and 250 mW, respectively. The minor and major axes of deformed cells are along *x* and *y-*directions, respectively. Each cell image is aligned with a black solid corner on the bottom left, and the vertical black (red) dashed lines represent the right cell boundaries of at the power of 150 mW (250 mW). (**c**) The dependence of relative deformation of the trapped human breast cells on the optical power. The relative deformation of the MCF10A PTEN-/- cells is around 6 times larger than that of MCF10A ones at the same optical power.

We used the AFMOTs to trap and deform individual MCF10A and MCF10A PTEN-/- cells, with each cell undergoing two successive loading/unloading cycles by changing the optical power. The results of the cell deformation by the optical trap are shown in Fig. 2. We moved the AFMOTs around to the desired cells at their initial locations. Each cell was first trapped by AFMOTs in three dimensions with a low optical power of 30 mW. The optical power was then increased and decreased between 30 and 250 mW to apply two cycles of optical force loading and unloading. At each power, we kept the optical power to be constant for at least 20 s before capturing the image to ensure a stabilized cell deformation. Typical images of a MCF10A cell and a MCF10A PTEN-/- cell under different optical powers are shown in Fig. 2a,b, respectively. It is clear that the MCF10A PTEN-/- cell (Fig. 2b) was deformed much more at the same optical power. Moreover, both cells were elongated along the *y*-direction (in the fiber plane) and shrunk in the *x*-direction (normal to the fiber plane). We repeated the loading/unloading experiment for four MCF10A PTEN-/- and four MCF10A cells, and the relative deformation (defined in Methods) is summarized in Fig. 2c. The uncertainty of each data point in Fig. 2c is determined by the standard deviation of the deformation of 4 cells. It can be seen that the cell deformation changes monotonically with optical power and, more importantly, that the deformation of MCF10A PTEN-/- cells is significantly larger than that of MCF10A cells. According to the results shown in Fig. 2c, we estimated the deformability (defined in Methods) of MCF10A PTEN-/- cells is 6 times that of MCF10A cells. With such a large difference, MCF10A PTEN-/- and MCF10A cells were faithfully distinguished when interrogated by AFMOTs, which indicates a great potential of AFMOTs to assist point-of-care disease diagnosis. In addition, this freedom of movable trap combined with the modular fiber trapping system allows AFMOTs to potentially find applications that are difficult for other fiber optical tweezers, such as biomechanical investigation of cells over a long time at their original locations in the cultured media or tissues.

Compared with traditional OTs, AFMOTs with cleaved fibers are a better candidate to deform cells of tens of micrometers in size. The main reason is that forces generated by AFMOTs are distributed over a large illuminated area of cell surface within the spot size, which is around 10 μm. By comparison, the trap of traditional OTs is at a diffraction-limit spot on the order of sub-μm. In order to achieve the same total optical force to deform cells, the optical stress and required intensity by AFMOTs are much smaller than those by traditional OTs, although the optical power is similar. Therefore, the AFMOTs promise to be a safer tool for cells, because the photodamage is linearly dependent on the optical intensity^47^. When higher optical forces are required to deform cells, AFMOTs allow a larger increase in power before possible photodamage occurs. As another important advantage, AFMOTs can deform cells without the help of any force handle or surface treatment, while beads are attached to cells as force handles by most traditional OTs for cell mechanics study^48,49^. No bead attachment is preferred because of the following reasons. First, the physical contact may introduce contamination and undesired physical or chemical modifications to cell surfaces. Secondly, optical forces applied onto beads give rise to concentrated forces applied on the cell membranes, which can develop nonlinear and non-uniform membrane stress distribution and in turn bring challenges to stress characterization induced by the bead-mediated point loading^50^. In addition, these concentrated forces are more likely to cause physical damage and disruption of cells than distributed forces^51^. By comparison, the AFMOTs applies distributed forces directly on cell membranes without any physical contact or additional force handles, which can minimize the cell disruption and allow cells to deform more evenly. With all the aforementioned reasons, AFMOTs are a unique tool that is safer and more suitable for cell mechanics study than traditional OTs.

**Figure 3:**
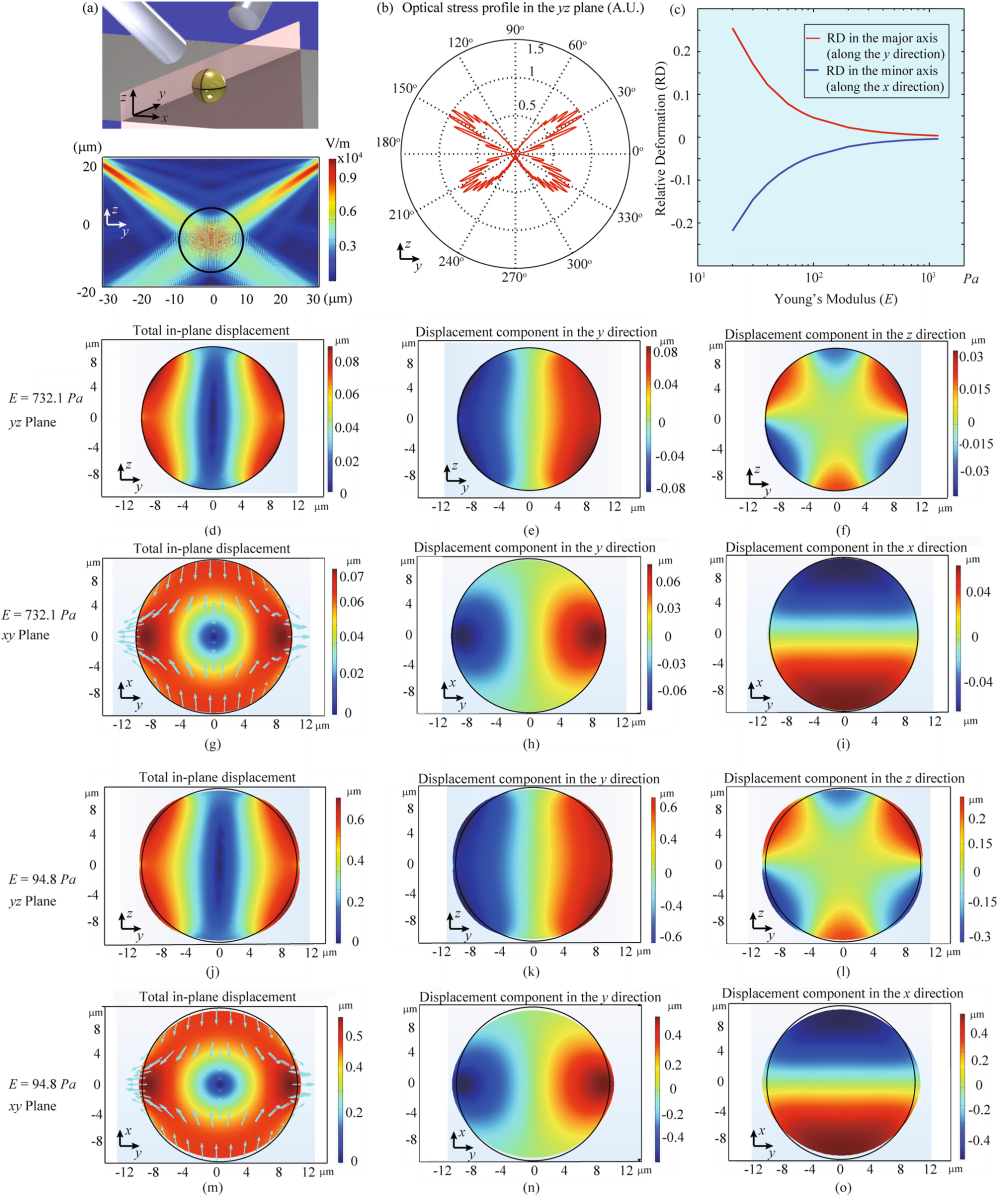
Simulation of cell deformation by AFMOTs. (**a**) (top) Schematic of the simulation model. A spherical cell with a diameter of 20 μm is trapped by AFMOTs. (bottom) Electric field distribution in the *yz* plane. The color map represents the absolute value of the calculated electric field. (**b**) Normalized stress profile experienced by the cell. (**c**) Dependence of the calculated relative deformation of the sphere major axis (along the *y* axis, red curve) and that of minor axis (along the *x* axis, blue curve) on the Young’s modulus (*E*). (**d**–**o**) Calculated displacement fields (color maps) and deformed cell shapes (profiles) of the cell at different *E* values. The *E* values of 400 Pa (**d**–**i**) and 80 Pa (**j**–**o**) are chosen to mimic a MCF10A and a MCF10A PTEN-/- cell, respectively. In-plane displacements are plotted in the *yz* (**d**–**f**, **j**–**l**) and *xy* planes (**g**–**i**, **m**–**o**) The first column (**d**, **g**, **j**, and **m**) shows the total in-plane displacement fields, with separate displacement components shown in the second (**e**, **h**, **k**, and **n**) and third (**f**, **i**, **l**, and **o**) columns. The black circles in (**d**–**o**) represent the original shapes of the cell. The arrows in (**g**) and (**m**) are the deformed direction of the cell surface, and the arrow magnitude is proportional to the displacement with a scale factor of 40 and 5, respectively.

We use commercial finite element analysis software (COMSOL Multiphysics) to model the cell deformation induced by optical forces in order to understand the experimental results. The simulation model is shown in Fig. 3a, where a cell is modeled as a solid sphere^51^ and the optical beam parameters are the same as those in the experiment (details in Methods). The optical stress on the cell surface is calculated and shown in Fig. 3b. We study the dependence of cell deformation on the cell Young’s modulus (*E*), with the results shown in Fig. 3c. The absolute value of the relative deformation decreases monotonically when *E* increases. By comparing with the experimentally measured relative deformation of the MCF10A cells in the *x* direction, we estimate their Young’s modulus to be 732.1 ± 265.3 Pa, which agree with those measured by other methods^52^. The detailed simulation results of cell profiles and displacement fields at *E* = 732.1 Pa are shown in Fig. 3d–i. We then calculate *E* of the MCF10A PTEN-/- cells in the *x* direction to be 94.8 ± 19.8 Pa, with the detailed simulation results of cell profiles and displacement fields at *E* = 94.8 Pa showing in Fig. 3j–o. The uncertainty of the *E* estimation is determined by the uncertainty of the experimental cell deformation at an optical power of 250 mW and the slope of the *RD*-*E* curve in Fig. 3c. The sources of the uncertainty include the cell movement in the recorded video and the algorithm for cell outer boundary fitting. The estimated *E* of MCF10A cells is ~ 7.7 times that of MCF10A PTEN-/- cells. This difference in *E* gives rise to the six times larger deformability of MCF10A PTEN-/-. cells observed in the experiment.

These simulation results confirm that AFMOTs not only provide faithful measurements of cell deformability and can differentiate single cells according to their mechanical properties, but also can be used to estimate the *E* of the measured cells. Combined with the modular and portable design, this capability bestows on AFMOTs a great potential for single-cell level disease diagnosis both in research labs and in point-of-care applications.

### 3D trapping of particles by AFMOTs

In addition to interrogating the mechanical properties of human breast cells, AFMOTs are a useful tool for 3D trapping of various particles based on optical fibers. By using different types of fibers in the same design of AFMOTs, we demonstrated three modalities that each have their own benefits and drawbacks. We will elaborate on the trade-offs and provide guidelines on how to choose from these modalities.

**Figure 4:**
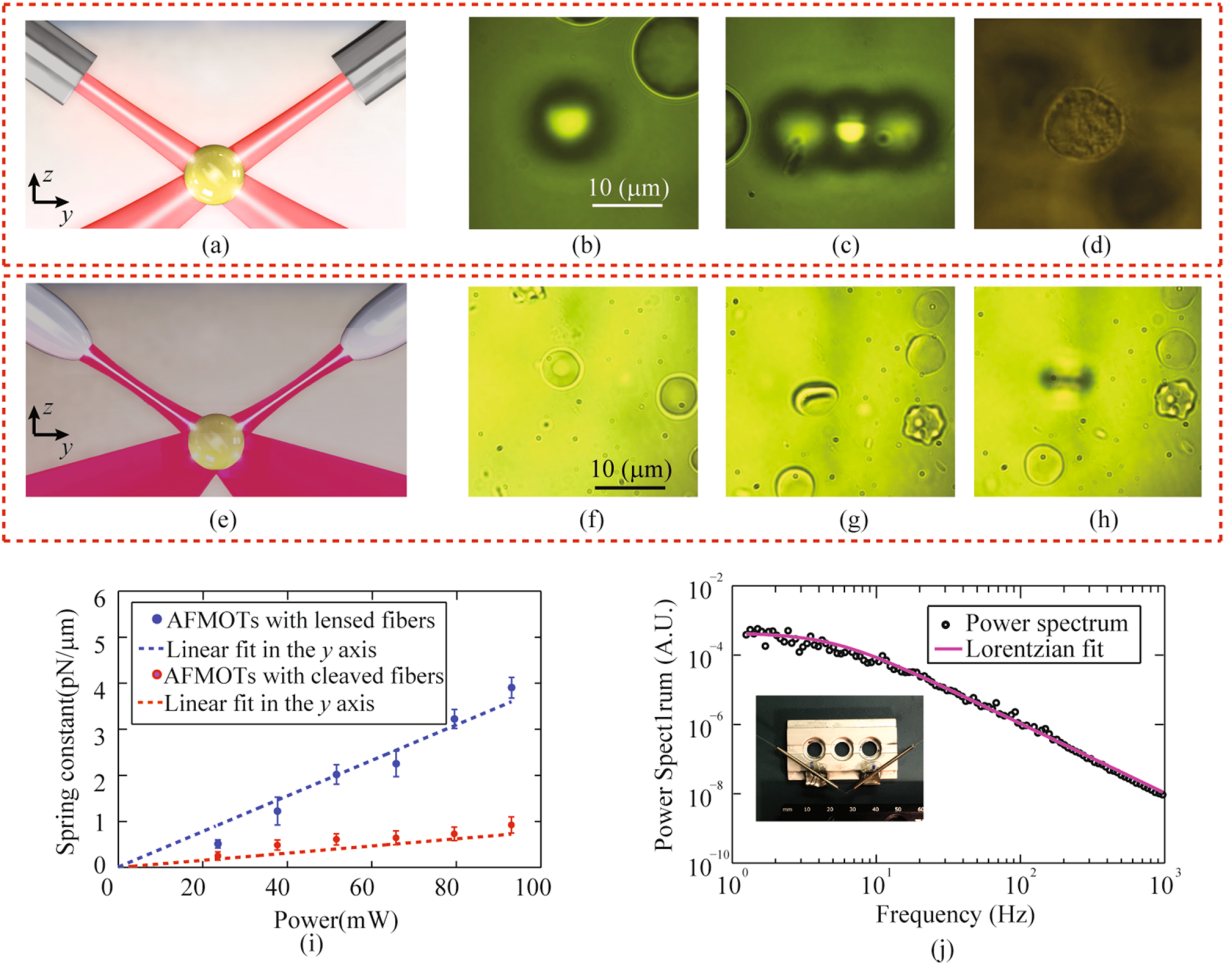
Demonstration of 3D trapping capabilities and the spring constant calibrations of two modalities of AFMOTs. The schematics of 3D trapping of AFMOTs with cleaved (**a**) and lensed fibers (**e**), respectively. A single (**b**) and multiple (**c**) polystyrene beads with a size of 15 μm are trapped in three dimensions by AFMOTs with single-mode cleaved fibers. The free beads lying on the cover glass have clear boundaries (in focus), while the blurry images of beads in (**b**) and (**c**) represent trapped beads (out of focus). (**d**) A spherical-like fibroblast cell stably trapped by AFMOTs with cleaved single-mode fibers. The dark shadows represent free cells lying on the cover glass. (**f**) A free rat red blood cell lying on the coverglass. This red blood cell was trapped, aligned along the *y* axis (**g**), and lifted above the coverglass (**h**) by AFMOTs with lensed single-mode fibers. (**i**) Optical trapping spring constant as a function of optical power based on the AFMOTs with cleaved fibers (red dots) and that with lensed fibers (blue dots). The extensions (red and blue dashed lines) of linear dependency of spring constant on optical power passes through point (0, 0). The optical power shown is the power emitted by each fiber. (**j**) Typical experimentally measured power spectrum data of a 15 μm bead trapped by lensed fiber AFMOTs in water (black circles) and Lorentzian fitting (solid purple curve) at 90 mW in the *y* axis.

Single-mode laser beams are commonly used in optical trapping because they provide excellent stability of the trap^16^. A single-mode fiber reliably delivers a fundamental mode by filtering out higher-order modes and isolating environmental fluctuations, while a cleaved fiber end extends its accessibility and cost-efficiency. The modality of AFMOTs with cleaved single-mode fibers (Corning HI 1060 in our work) combines all the aforementioned benefits, finding a good balance between the trapping performance and the system cost. Figure 4a shows the schematic of 3D trapping of AFMOTs with cleaved fibers. In addition to trapping and deforming human breast cells, we demonstrated the capabilities of this modality by trapping single and multiple polystyrene particles, as well as floating fibroblast cells, as shown in Fig. 4b–d. Practically, multiple particles can be trapped due to optical binding effects, as shown in Fig. 4c. The smallest cells we successfully trapped are around 8 μm in diameter, including human breast cells and fibroblasts.

A lensed single-mode fiber has a larger numerical aperture (NA) (0.4 for TLF SM1060, see Methods) than that of cleaved fiber (0.14 for cleaved HI 1060). Laser beams with a higher NA help increase the trapping efficiency and trapping stability, and allow smaller particles to be trapped in three dimensions. The schematic of 3D trapping of AFMOTs with lensed fibers is shown in Fig. 4e. In addition to all the cells and particles trapped by cleaved fiber AFMOTs, the modality with lensed single-mode fibers was used to trap rat red blood cells of around 4 μm in diameter, as shown in Fig. 4f–h. The smallest particles we have successfully trapped in 3D are 3-μm-diameter silica beads. This modality has a considerably enhanced trapping efficiency (see the calibration results below) and stability, making it ideal for applications involving small (3–8 μm) particles and those with high requirements on trapping performance. The downside is the compromised accessibility and cost. It is difficult to fabricate lensed fibers repeatably and controllably. Although lensed fibers can be obtained commercially, the high price and long lead time could limit the applications of this AFMOTs modality, compared with that with cleaved fibers.

Telecommunication fibers, especially single-mode fibers at 1.3–1.6 μm, are particularly interesting to build AFMOTs, due to the unparalleled accessibility, cost efficiency, and availability of compatible devices. Using the AFMOTs built with cleaved Corning SMF-28 fibers, all the 3D trapping results in Fig. 4b–d were repeated. Although SMF-28 fibers are in the multimode regime at our laser wavelength of 974 nm, the measured spot size and the intensity profiles at the trap are similar with those from cleaved HI 1060 fibers. It is noted that the modality of cleaved SMF-28 fibers could suffer from the inferior position and optical stability of the trap due to the presence of higher order modes in the fibers. However, we did not observe any instability issues in our experiment, while similar cell deformation results, as shown in Fig. 2, have been obtained. As a result, the combination of the SMF 28 fibers and the modular design make the AFMOTs with cleaved SMF-28 fiber excel in the cost and accessibility and even more attractive, especially considering that the 3D trapping and detection (detailed later) capabilities are retained.

### Objective-lens-free position detection and spring constant calibration

In addition to the 3D trapping, AFMOTs have the capability of nm-resolution particle position detection (see Methods), which is enabled by the same two optical fibers used for trapping, free of the microscope and objective lens. The calibration of optical spring constant can hence be performed based on the power spectrum analysis (details in the Methods). It is noted that the fiber-based calibration is available in all three modalities of AFMOTs, namely the AFMOTs based on cleaved single-mode fibers, lensed single-mode fibers, and cleaved SMF-28 fibers. When a 15-μm polystyrene bead was trapped in water, the experimentally obtained calibration results are shown in Fig. 4i. A linear relationship exists between the spring constant and power for both the AFMOTs with cleaved HI 1060 fibers and that with lensed TLF SM1060 fibers. The linear fitting provides the corresponding normalized spring constant to be 7.5 pN/μm/W and 37.5 pN/μm/W, respectively. The higher normalized spring constant of the AFMOTs with lensed fibers arises from the higher NA of the optical beam. A typical set of power spectrum data in the *y*-axis are shown in Fig. 4j, from which we obtain a corner frequency of 4.92 ± 0.28 Hz and the corresponding spring constant of 3.89 ± 0.22 pN/μm. The fiber-based calibration can also be carried out in the AFMOTs with cleaved SMF 28 fibers (results not shown), and the spring constant is comparable to that with cleaved HI 1060 fibers.

The demonstrated capabilities of simultaneous trapping and fiber-based detection are particularly attractive in the portable, modular configuration of AMFOTs, which can bring the optical trapping technology to practical applications in the field.

## Discussion

This paper presents a miniature and integrated fiber optical trapping system, namely AFMOTs. The modular design not only eliminates the maintenance of fiber alignments and free-space optics, but promises to be mechanically robust and reliable for long-duration applications. We have observed an accumulative fiber misalignment of < 5 μm in AFMOTs over 2 months. Further research to extend this period is underway. In addition, the AFMOTs retain the capabilities of optical trapping, manipulation, and detection of the trapped particle positions, all based on optical fibers. As a demonstration of the AFMOTs capabilities and benefits, we differentiated diseased human breast cells from healthy ones. The system can be even more versatile by using different types of fibers to adapt to various applications.

Some trapping performances of the AFMOTs are similar with the non-modular dual-fiber tweezers^41,42^, such as the spring constant differences along the *x* and *y* directions. The AFMOTs have great potential to bring the optical trapping technology out of the lab and to develop portable tools for practical applications.

There are some limitations in AFMOTs, with the most notable ones listed below. (1) It is difficult to trap particles deeply (> 100 μm) inside a 3D solid compartment because the fiber tip separation is relatively small (~ 150 μm, see Method) and the trap is relatively close to the fiber tip. However, in a liquid medium, the trap can be freely moved anywhere in the solution by moving the modular AFMOTs. (2) The fiber-based detection in AFMOTs can be used to detect one-dimensional (*y* direction) displacement of the trapped particle, without any coupling with the other two dimensions. In applications where more than one-dimensional measurements are required, such as in anisotropic media, the AFMOTs can be rotated by 90º to measure the particle motion in the orthogonal directions. (3) The fiber-based detection mechanism in AFMOTs enables faithful measurements of the trapped particle position, but not the particle shape changes. The measurements of the shape changes of biological samples still need an objective lens and a camera.

It is noted that the speed of the cell deformability measurements demonstrated in this work is much lower compared with that by the optical stretcher^53^. We would like to point out that the demonstrated speed is nowhere close to the speed limit. The slow speed resulted from the loading/unloading cycles and moving the AFMOTs to different locations. We chose the loading/unloading cycles in our cell experiments to illustrate that the AFMOTs induced cell deformation was recoverable and repeatable. The AFMOTs was moved to each cell’s original location in the media in order to demonstrate the trap movability. Similar to the optical stretcher, the two inclined fibers in AFMOTs can also be integrated with microfluidic channels by making the channel substrate as the common board. Such an integration gives up the mobility of the trap, but a similar throughput of the cell measurements can be expected. Since both the optical stretcher and AFMOTs are modular fiber optical tweezers, we would elaborate on the difference between these two systems, which is the mobility of the trap. The two fibers in the optical stretcher are aligned along the same line and integrated with a substrate, which is generally the same as the substrate of microfluidic channels^21,53,54^, to maintain the fiber alignments. As a result, the optical trap cannot move freely, and cells to be tested need to be fed into the trap by fixed microfluidic channels. By comparison, the trap location in the AFMOTs is independent of the cell substrate, and cells in an open channel or on an un-patterned surface can be readily trapped by AFMOTs. Such a mobile trap allows AFMOTs to find applications that are difficult for optical stretchers, such as studying the deformability of cells on a cover glass and moving a cell lying on a tissue surface.

The AFMOTs are a self-sustaining, portable, and integrated tool particularly useful for biomechanics studies. Compared to the artificial corruption using standard genomic or proteomic techniques, which are commonly used for biochemical disease diagnosis^55^, AFMOTs provides a label-free, mechanical method for single-cell-level disease diagnosis, with minimum requirements for the cell substrates. In addition to healthcare applications, the low cost of AFMOTs grants high accessibility that allows the optical trapping technology to reach the masses, especially to K-12 and higher education.

## Material and methods

### Optical system and implementation of the AFMOTs

The optical system of AFMOTs is similar to the dual-fiber optical tweezers, which can be found in our previous works^41,42,56,57^. Briefly, AFMOTs were mounted onto a 3D translational stage (green block shown in Figure S1a) with a travel range of 1″ in each direction. Light from a fiber-coupled 974 nm laser diode (AC 1405-0400-0974-SM-500, Eques) was split into two pre-fixed fibers through a 3 dB coupler (22-12798-50-23162, Gould Fiber Optics), as shown in Figure S1a. The two light beams emitted from fibers can three-dimensionally trap particles with different sizes in liquid media close to the beam intersection. The back-scattered light by the trapped particle was collected by the same two fibers and in turn, enabled the nm-resolution particle position detection (see “Fiber-based detection and calibration”). The cell deformation experiment was carried out on a Nikon Ti-U inverted microscope. The AFMOTs were mounted on a 3D stage next to the body of the microscope and the trap was moved to the view field of the microscope objective lens. The images of trapped particle deformation were collected by an oil-immersion objective lens with a numerical aperture of 1.4, was recorded by a C-mount microscope camera.

### Fibers used in the AFMOTs

Various types of fibers can be used in AFMOTs. In our experiment, we have used 3 fiber types, which are the cleaved single-mode fibers at 974 nm (HI 1060, Corning), the lensed single-mode fibers at 974 nm (TLF SM1060, Nanonics Imaging Ltd), and the cleaved multimode fibers at 974 nm (SMF 28, Corning). The experiments, including the interrogation of cell deformability (Figs. 2, 3) and the optical trapping of spherical particles (Fig. 4b–d), were carried out by AFMOTs with cleaved single-mode HI 1060 fibers. AFMOTs with lensed single-mode fibers were used to trap red blood cells from a rat. Optical trap calibration, as shown in Fig. 4i, j, was carried out by fiber-based detection (see “Fiber-based detection and calibration”) using the AFMOTs with cleaved HI 1060 and lensed TLF SM1060 single-mode fibers, respectively. It is noted that experiments of 3D trapping, cell interrogation, and optical trap calibration from AFMOTs with single-mode cleaved HI 1060 fibers were also carried out in the AFMOTs with SMF 28 fibers (results not shown), and their results are comparable.

### Fiber-based detection and calibration

Fiber-based position detection of trapped particles can be used to calibrate the spring constant of the optical trap. According to our previous work^36^, the differential signals (scattering light) collected by two fibers in AFMOTs scale with *y*-displacement (Fig. 1c) relative to the trap center when the motion of the trapped particle is small (< 100 nm). Once a particle is trapped, no objective lens or microscope is required to detect the particle position. In the experiment, the back-scattered light by the trapped particle was collected by the two prefixed optical fibers and measured by two inputs (PD1 and PD2) of a balanced photodiode (PDB450C, THORLABS). AFMOTs can achieve 2 nm spatial resolution and hundreds of MHz bandwidth.

### COMSOL simulation

In the simulation, we model a human breast cell as an incompressible sphere with a refractive index of or 1.4^58^ and a Poisson’s ratio of 0.5^59^. The cell deformation is calculated in two steps. In the first step, we calculate the optical stress distribution in the *yz* plane (see Fig. 3a) by inputting the same parameters (average cell size, optical power, spot size, and optical intensity distribution) as those in the experiment. In the second step, we use the optical forces obtained from Step 1 as an input parameter to calculate the 3D deformation of the cell.

In Step 1, we calculate the optical forces and the corresponding stresses over the sphere surface within the illuminated spot size. In this step, a 20 μm sphere is surrounded by water in a domain that is surrounded by perfectly matched layers. Two incident optical beams, with an inclined angle of 55°, are launched into the medium. Each light is set as a “port” input with an electric mode designed as a Gaussian function. Since the refractive index of the sphere is higher than that of the medium, the sphere experiences stretching optical stresses and forces. According to our simulation, the optical forces on the cell surface are up to hundreds of pico-Newton (~ 200 pN) when each light power is 250 mW in AFMOTs.

In Step 2, we apply on the cell surface the optical stresses, which are Gaussian distributed (supplemental information) over the illuminated sphere surface within the spot, with both the spot size and optical stress magnitude obtained from Step 1. The deformation of the sphere is studied as a function of Young’s modulus (*E*) of the sphere, with the results shown in Fig. 3c. The simulation model is symmetric about the *yz*-plane, resulting in no *x*-displacement in the *yz*-plane. It is noted that the cell relative deformation is obtained in the experiment from the microscope images, which are the *xy*-plane projections of the 3D deformed cells with the *z*-axis displacement information removed. Therefore, only in-plane displacements are plotted both in the *xy*-plane and *yz*-plane in the simulation (Fig. 3d–o).

The experimental results used to compare with the simulation are the relative deformation at 250 mW measured in the first cycle of loading/unloading. Particularly, the relative deformation of MCF-10A cell is 0.8%, and that of MCF10A PTEN-/- cell is 5% in the *x* direction.

### Algorithm for outer boundary detection of cells

Bright-field images of cells were obtained by a C-mounted camera through an oil immersion objective lens with a numerical aperture of 1.4. The images were cropped manually so that the field-of-interest was fairly filled by the cell. The cropped images were analyzed using a custom-built script in Matlab (Mathworks, Natick MA) to find its boundary. Eroding the image (within the cell’s edge) and using the *‘imfill’* command to fill the inside area of the cell, we obtained a binarized image with an elliptical shape. The lengths of the cell major or minor axes were extracted by fitting the binarized oval, and the deformation of the cells were calculated accordingly.

### Definitions of the relative deformation and the deformability of human breast cells in the experiment

In the experiment, we measured a 3D trapped cell at the lowest trapping power (30 mW) to determine its initial lengths of the cell major and minor axes. To quantify the deformation of MCF10A PTEN-/- and MCF10A cells, we define the relative deformation of a cell by the length change of its major (minor) axis normalized by the initial length. According to the dependency of relative deformation (*RD*) of human breast cells on power (Fig. 3c) in each loading/unloading cycle, we define the deformability (*DFM*) of human breast cells to be proportional to the slope of the *RD VS power* curve.

By averaging the slope in the minor axis in all optical loading/unloading cycles, we estimated the deformability of MCF10A and MCF10A PTEN-/- Cells, with the plot shown in Fig. 2c. Similarly, the deformability of both types of cells can also be obtained in the major axis.

### Definitions of the inclined angle and fiber tip separation in AFMOTs

In the experiment, the inclined angle θ and the fiber tip separation were important for AFMOTs. We define θ as the angle between fiber and the vertical direction, and two fibers share the same θ value. We define the fiber tip separation as the distance between the two fiber cores at the fiber end faces. In this work, we set the inclined angle and the distance between two fibers to be 55° and 80 μm, respectively. The influence of the fiber inclination angle and separation between two fibers on the trapping efficiency, trap stability, and minimum optical power needed for a 3D trapping can be found in the supplementary information.

### Cell line and cell culture

NIH-3t3 fibroblasts were cultured in the DMEM medium (BioWhittaker, Walkersville, MD) that was supplemented with 10% BCS growth serum (Gibco, Waltham, MA), 2 mM l-glutamine (Gibco), 100 µg/ml streptomycin and 100 units/ml penicillin (Gibco).

The cells used in the cell deformability measurements are the MCF10A human breast epithelial cell line and the MCF10A PTEN-/- cell line, a cancerous mutant cell line of MCF10A lacking the tumor suppressor gene, PTEN. MCF-10A and MCF10A PTEN-/- cells were gifts from Dr. Michele Vitolo at the University of Maryland. Cells were cultured on tissue culture plastic in a 1:1 mixture of Dulbecco’s modified Eagle’s medium and F12 medium (DMEM-F12) supplemented with 5% horse serum, hydrocortisone (0.5 g/ml), insulin (10 g/ml), epidermal growth factor (20 ng/ml), penicillin–streptomycin (100 g/ml) and 0.1 μg/mL cholera toxin.

### Preparation of the bead solution

Silica beads, with a diameter of 4.63 μm and a density of 2.0 g/cm^3^, and Polystyrene beads, with a diameter of 15 μm and a density of 1.2 g/cm^3^, were used in the experiments. Both types of beads were purchased from Bangs Laboratories, Inc. A bead solution was diluted with a ratio of 1:6000 (original bead solution (10%, 0.5 g in weight) to deionized water) and was ultrasonicated in order to reverse bead aggregation. A few drops of bead solution were then added on a coverslip, where the trapping experiment was carried out.

### Power spectrum analysis method

The optical trap was calibrated by power spectrum analysis method^60^. Fiber-based detection in AFMOTs provides the motion of a trapped particle, which was then Fourier transformed into the frequency domain. The spring constant of the optical trap was obtained by fitting the blocked power spectra to a Lorentzian function.

## Supplementary information

Supplementary Information
