# Supplementary material for: Reliable and mobile all-fiber modular optical tweezers
Source: Sci Rep. 2020 Nov 18;10:20099. doi: 10.1038/s41598-020-77067-1 (PMC7676252; doi:10.1038/s41598-020-77067-1)
Supplement: Supplementary file 1 — Supplementary Information [file 41598_2020_77067_MOESM1_ESM.docx]

Supplementary information

Reliable and Mobile All-fiber Modular Optical Tweezers

Chaoyang Ti ^1,*^, Yao Shen ^1^, Minh-Tri Ho Thanh^2^, Qi Wen^2^, and Yuxiang Liu^1, 2^

^1^Department of Mechanical Engineering, Worcester Polytechnic Institute, Worcester, MA, 01609, USA

^2^Department of Physics, Worcester Polytechnic Institute, Worcester, MA, 01609, USA

*Correspondence to (yliu11@wpi.edu)

This document provides supplementary information to “Reliable and Mobile All-fiber Modular Optical Tweezers". It includes more details on (1) Fabrication of the AFMOTs, (2) Influence of the inclination angle and separation between two fibers, (3) Simulation of the optical field of AFMOTs, (4) Optical trapping of Human Breast Cells, and (5) Observation of Cell photodamages: membrane blebbing and rupture.

1. **Fabrication of the AFMOTs**

Fabrication of the AFMOTs was realized by permanently affixing two pre-aligned optical fibers onto a common board, as shown in [Figure S1(b)], which enables the optical trapping system to be used without any further alignments of fibers and to be delivered outside a lab environment. Briefly, the fabrication process includes supporting the fibers by metal tubes, fiber pre-alignments, transfer to a common board, fixation of fibers on the common board, and release of fiber holders. The fabrication process is the same for different fibers discussed in the main text. These steps are explained in detail below.

The optical system used in both system fabrication and trapping experiment was shown in Figure S1(a), similar to that used in our previous publication[^1^](#_ENREF_1), detailed in **Optical system and implementation of the AFMOTs** in Methods. To reduce the mechanical drift and vibration of fibers in AFMOTs, we inserted each fiber into a stainless-steel tube with the inner and outer diameters of 270 μm and 1.5 mm, respectively. The suspending length of the fiber tip outside the tube was ~4 mm, as shown in Figure S1(b). A UV glue (NOA68, Norland Products, Inc.) was applied on both ends of each metal tube and cured to secure the fiber.

The pre-alignments of fibers are based on the quality of the laser beam (or laser spots) emitted from the two fiber tips. Each tube was held by a fiber holder mounted on a set of translational and rotational stages for position and orientation adjustment. As a coarse alignment, we adjust the two sets of stages to ensure that the intensity profiles of the two laser spots are close to each other in the same view field of the microscope camera. The two fibers were then aligned to limit the *x*-axis and *z*- axis misalignments of less than 1 μm and 2 μm, respectively. The fiber alignment procedure is illustrated in Figure S2(a)-(c). Particularly, the *x*-axis misalignment influences the trapping stability the most and hence is the most important parameter to control in the alignment. We determined this parameter by the *x*-axis distance between the two spots in microscopic images. Figure S3 shows a typical microscopic image of two well-aligned spots with less than a 1-μm *x*-axis misalignment. The yellow and red curves are raw intensity profile and polynomial fitting, respectively, along the dashed white line of the image. The *y*-axis fiber separation (defined in the Methods section of the main text) and fiber inclination angle are two design parameters determined by the sample sizes and the desired system performance (beam spot size, trap strength, total system size, etc.), and will be discussed in more details in the next section. In this work, the *y*-axis fiber separation was fixed to be 80 μm and the inclination angles of both fibers were 55°, following those used in our previous publication[^1^](#_ENREF_1).


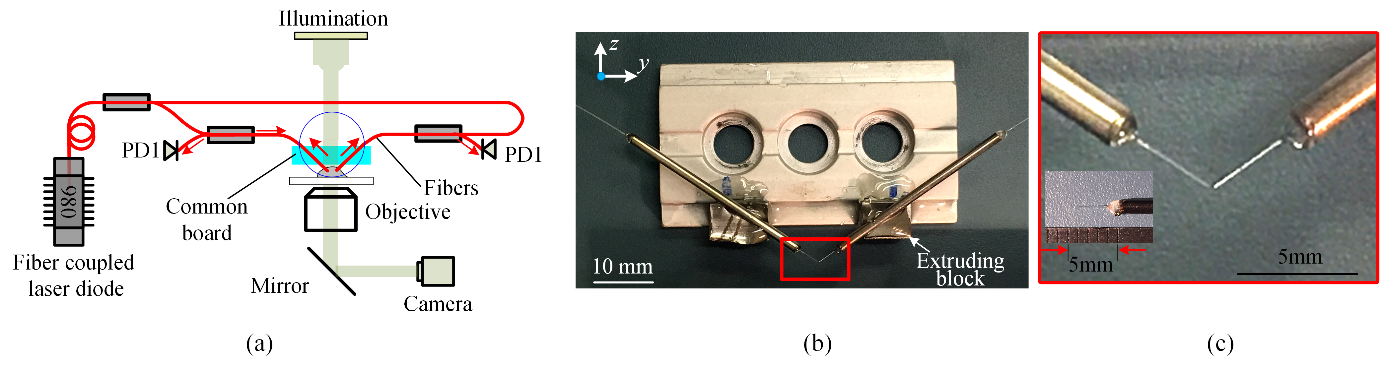

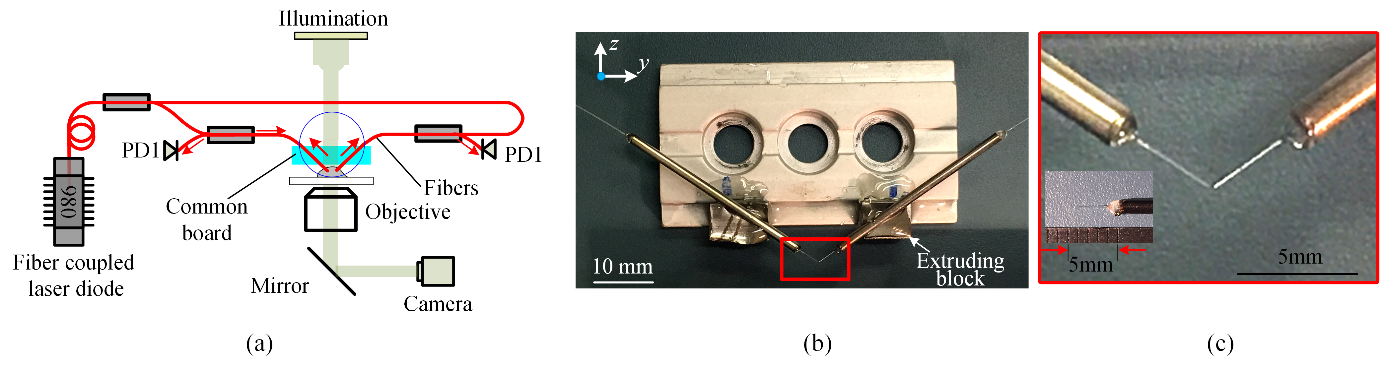


Fig S1 (a) schematics of the experimental setup of AFMOTs with the fiber detection mechanism. The two fibers in AFMOTs are used to trap particles and detect the trapped particel postion, which in turn used to sense the optical forces provided by the AFMOTs . Its close-up image, blue circle in (a), are shown in (b). PD1 and PD2 are the two optical inputs of the same differential photodiode. The camera is used to check the quality of the alignment of AFMOTs. (b) photographic image of AFMOTs. Two fibers permanently aligned on a common board via two extruding blocks in the *yz* plane. Two extruding blcoks are mounted at the bottom of common board. (c) The close-up view, red rectangle in (b), of AFMOTs. Inset: A cleaved fiber inserted into a stainless-steel tube and secured by UV glue (semi-sphere at the tip of stainless-steel tube). Its suspending length is ~ 4mm.


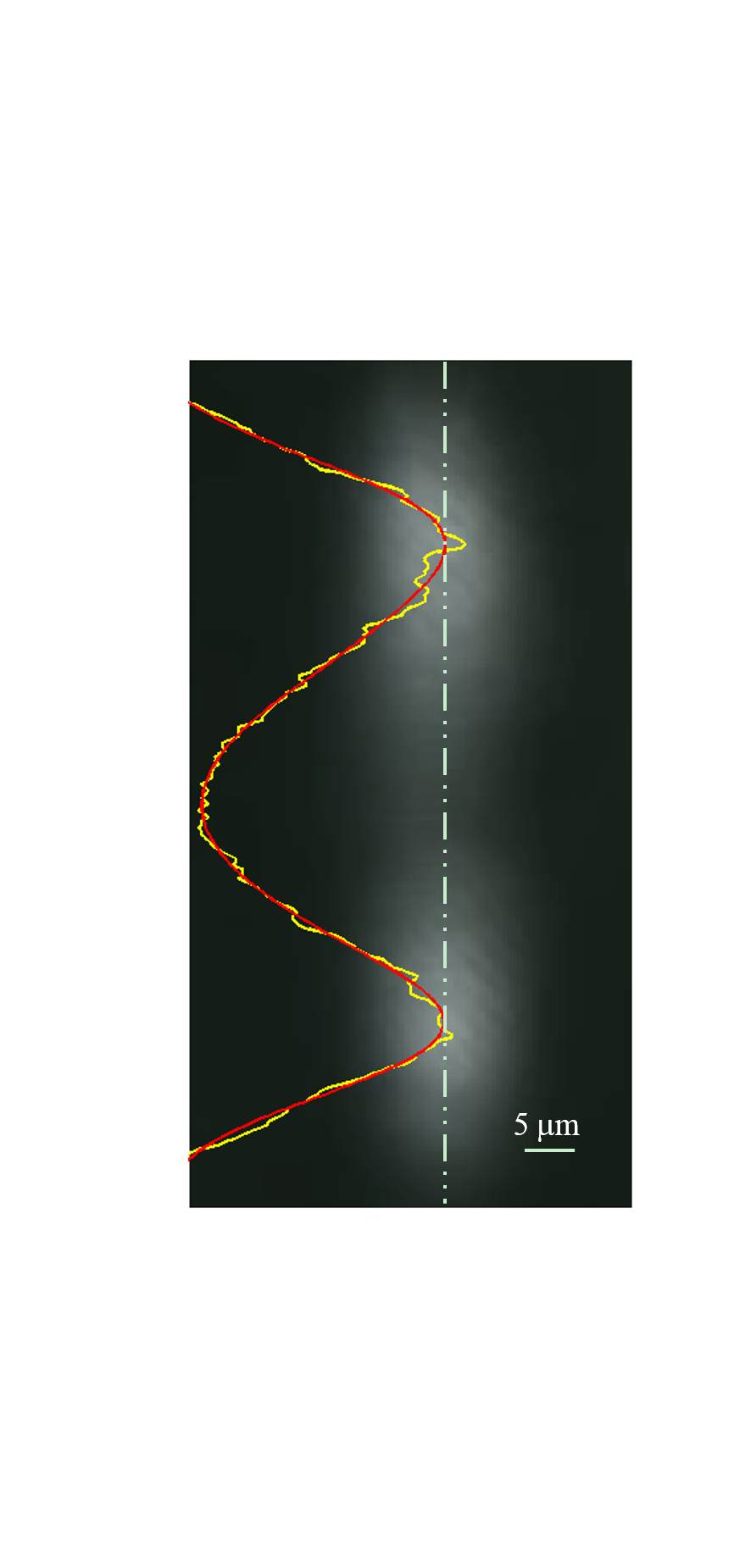


Fig S3 Microscope image of the light spots emitted from the two fiber ends during the fiber alignments of AFMOTs. The yellow and red curves are the raw data and filtered curve, respectively, of the intensity distribution along the white dot-dashed line.

After the fibers were aligned, we trapped a silica beam with a size of 4.63 μm and brought a common board toward the fibers by another translational stage for the transfer process (Figure S2(d)). The common board is an aluminum board with a total size of 1/8" x 1/4" x 1" with three through holes, which allow the final AFMOTs to be conveniently mounted on a wide range of platforms, such as microscopes and motion stages, by regular screws. Two steel blocks, 10 mm x 6 mm x 5 mm each, were glued on the bottom of the common board and created two plateaus to fix the fiber tubes, as shown in Figure S1(b). We pre-attached the UV glue (NOA68, Norland Products, Inc.) onto each flat surface of the steel block. The common board is moved until one of the flat surfaces touching one of the fiber holders. We cure the UV glue to fix it on the common board first. Noted that there might be a gap between the other fiber and steel block. However, the UV glue is highly viscous and enough to cover this gap. We then cure the UV glue on the other steel block. The optical trapping, used to monitor the stability, is still maintained during the curing procedure. The fibers were then released from the fiber holders (Figure S2(e)), rendering a modular AFMOTs with fibers well aligned and fixed (Figure S2(f)).


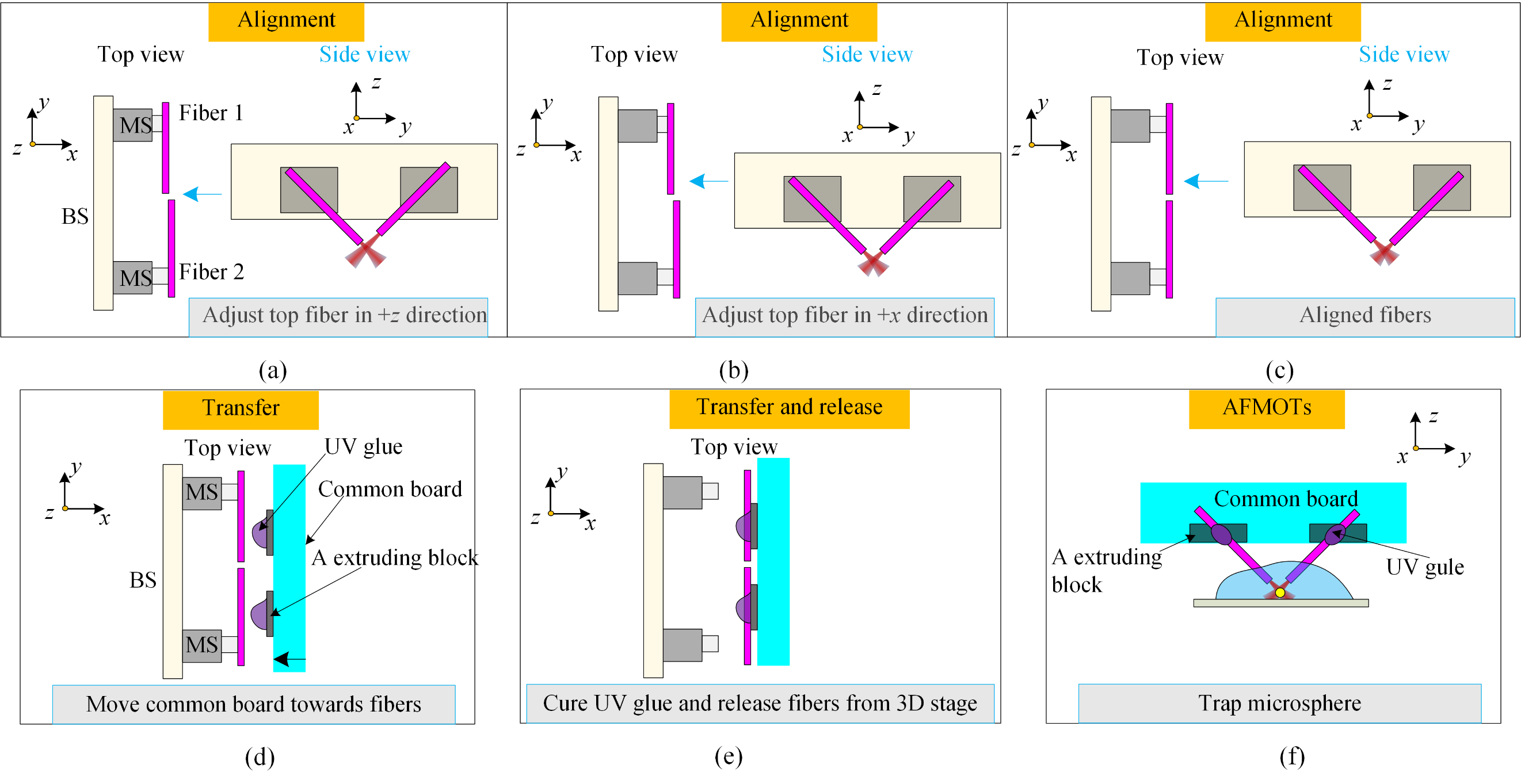


Fig S2 Schematics of AFMOTs’ fabrication, including procedures of fibers aligning (a)-(c), fibers transferring (d), and fibers releasing (e). Top view of two fibers (pink bars) mounted on two sets of micro-stages with the top fiber adjusting along + *z* (a, left) and +*x* (b, left). Top view of aligned fibers in (c, left). The arrows (blue) in (a-c) point the side view direction and the corresponding side views are shown in (a)-(c), right). (d) Two aligned fibers transferred to a common board with extruding blocks attached. High viscous UV gule was pre-attached on the extruding blocks. The black arrow shows the direction of movement of the common broad. (e) Two aligned fibers released from two sets of microstages. (f) Schematics of optical trapping of AFMOTs. Yellow bars on the top and the blue frames at the bottom in figures (a)-(f) stand for the representative fibrication procedures and the detailed operations in their procedures, respectively. BS: big stagte with a common board; MS: micro stages.

1. **Influence of the fiber inclination angle and separation between two fibers**

According to our previous work[^2^](#_ENREF_2), the inclination angle can influence the trapping efficiency, trap stability, and minimum optical power needed to realize the 3D trapping. Generally, the larger the inclination angle, the higher trapping efficiency, and less minimum optical power needed to realize the 3D trapping. However, a larger inclination angle increases the alignment difficulty and decreases the trapping stability. The *y*-axis fiber separation determines the trapping efficiency of AFMOTs and influences the size range of the samples that can be trapped and deformed. The trapping efficiency is defined as the ratio of optical spring constant to optical power. The larger the y-axis fiber distance, the smaller the trapping efficiency of AFMOTs. Moreover, a larger *y*-axis fiber distance results in larger spot sizes of the trapping beams, which help to trap and deform larger biological cells. A more detailed discussion of the setup parameters can be found in our previous work[^1^](#_ENREF_1), which has a similar setup of included dual fibers but is not a modular system.

1. **Simulation of the optical field of AFMOTs**


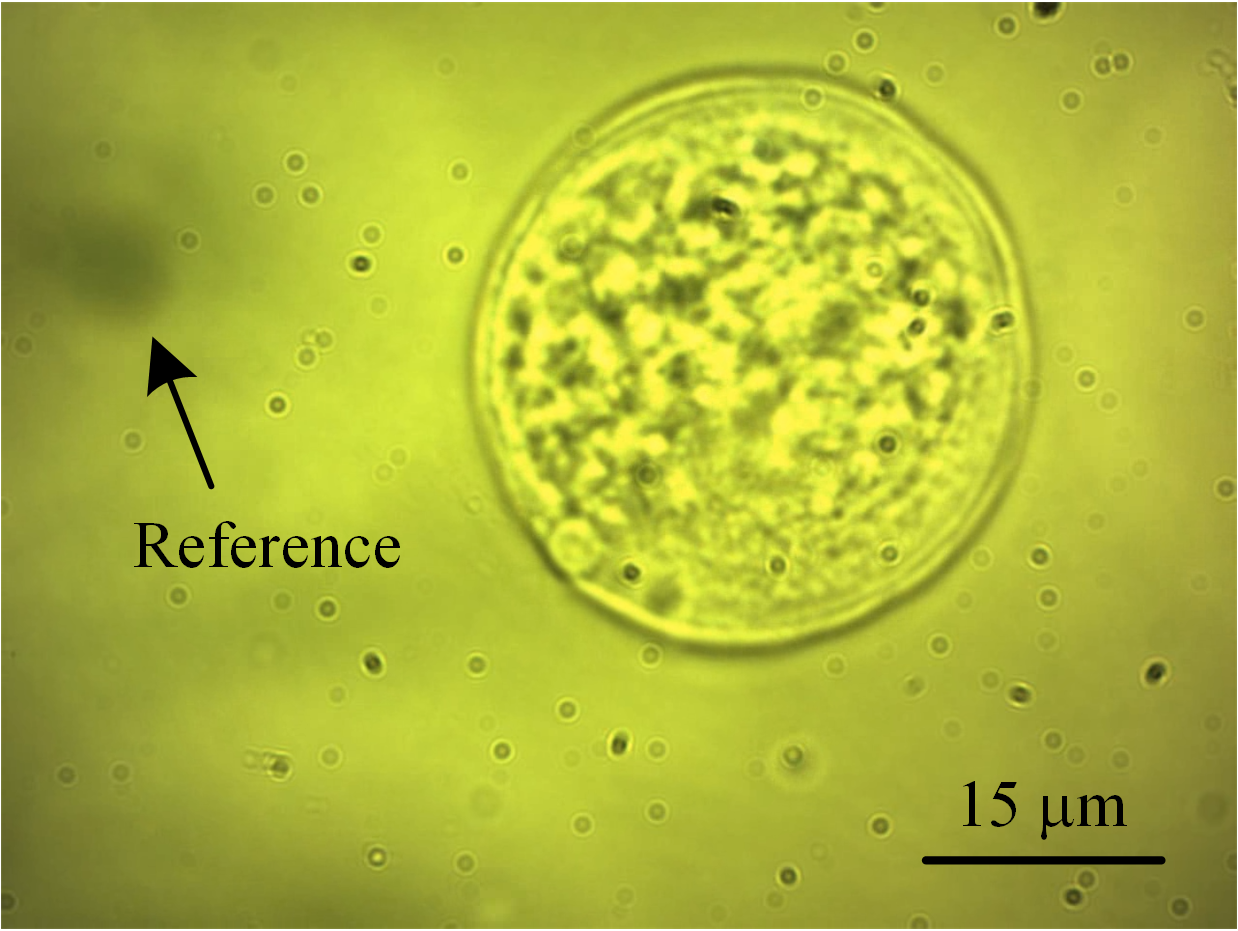


Fig S5 Demonstration of the 3D trapping capability of AFMOTS. A breast cancer cell with a size of ~32 μm is trapped by AFMOTs. The black arrow points to the reference breast cell lying on the cover glass. The reference is out of focus because the trapped cell was lifted away from the cover glass.

We used COMSOL Multiphysics to numerically study the trapping performance of AFMOTs. In the simulation, all parameters, such as the fiber inclination angle and fiber distance, are the same as those in the experiments. The fiber type used in the simulation is SMF 28, which was used in the cell deformation experiment.


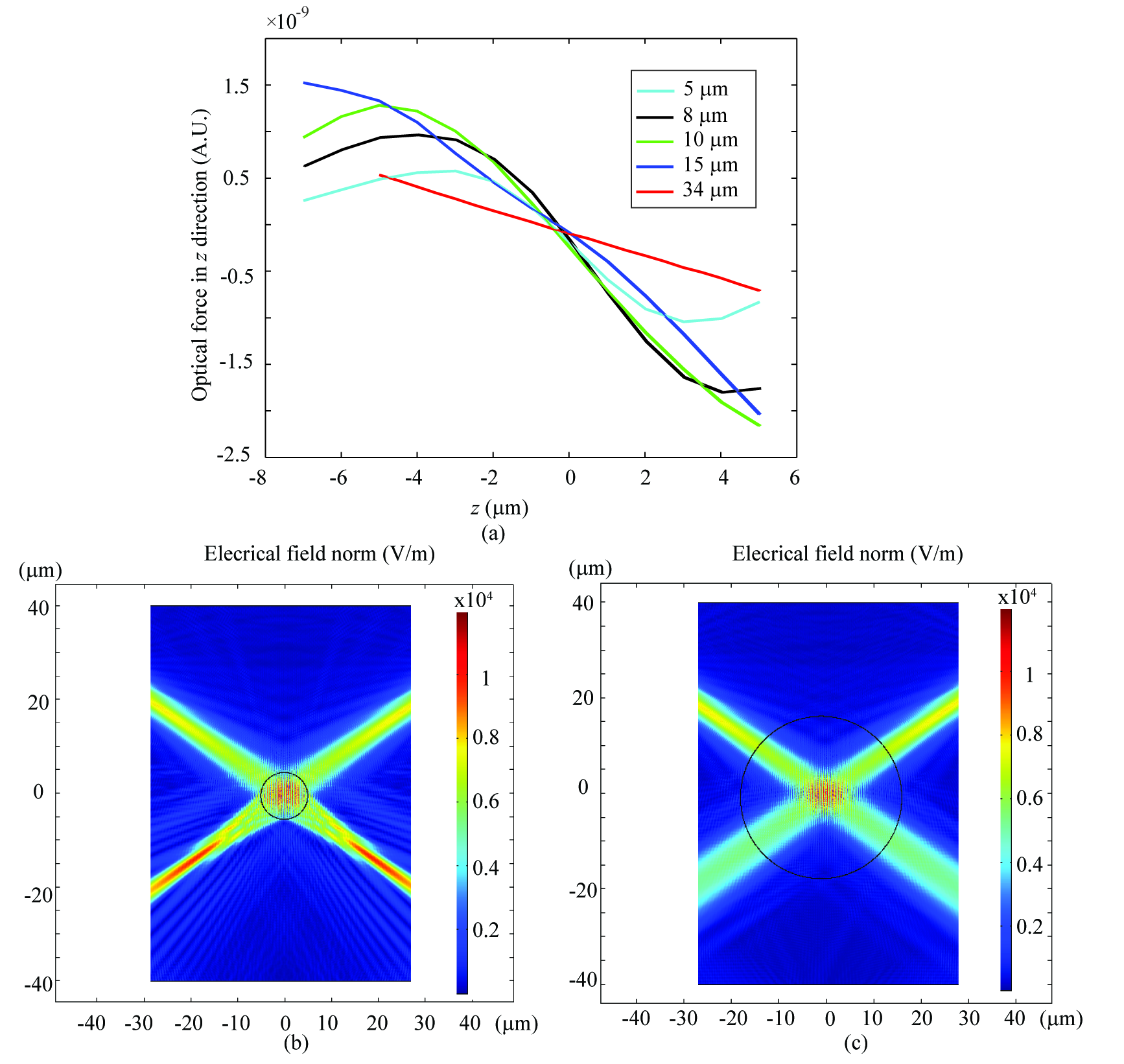


1. (b)

Fig S4 Simulation results of the electrical field magnitude in the *yz* plane (see Figure S1 for the coordinate system) when the emitted lights from AFMOTs pass through a silica sphere with a diameter of 10 μm (a) and that of 34 μm (b).

Figure S4 shows the optical field distribution of AFMOTs with two different beads in the trap. We note that light can be focused and in turn formed an enhanced localized field gradient when passing through a small particle with a size of 8 μm (Figure S4(a)), while it is not focused by a large particle with a size of 34 μm (Figure S4(b)). As a result, multiple small particles with a size close to the light spot in the trap can be successfully trapped by AFMOTs due to the optical binding effect[^3^](#_ENREF_3). As shown in Figure 4c in the main text, the center bead is trapped and the other two side beads are located between the trapped bead and the substrate. However, we did not observe this optical binding effect in the cell experiments.

1. **Optical trapping of Human Breast Cells**

The human breast cells were pre-treated (see Method) to allow them to float in the medium. A few droplets of the diluted cell solution were added onto a cover glass, where the experiment was carried out. Once a breast cell (either an MCF10A PTEN-/-. or MCF10A cell) was trapped, as shown in Figure S5, it can be moved in *x*, *y*, and *z* dimensions by moving the common board of the AFMOTs. The working distance of AFMOTs is not confined to the vicinity of the cover glass, which is the case for traditional optical tweezers built with an oil-immersion objective lens. As a result, the trapped cells can be moved anywhere in the medium. We used the AFMOTs with cleaved fibers (SMF 28 and HI 1060) to successfully trap the breast cells with sizes ranging from 8 to 32 μm in three dimensions. Cells with a size smaller than 4 μm were able to be trapped by AFMOTs with lensed fibers (TLF SM1060), but not by those with cleaved fibers. We did not experimentally test cells larger than 32 μm for optical trapping with the AFMOTs. In the experiment, the size of the cell was determined by the length of its major axis (*y*-axis in (Figure 1 and 3)).


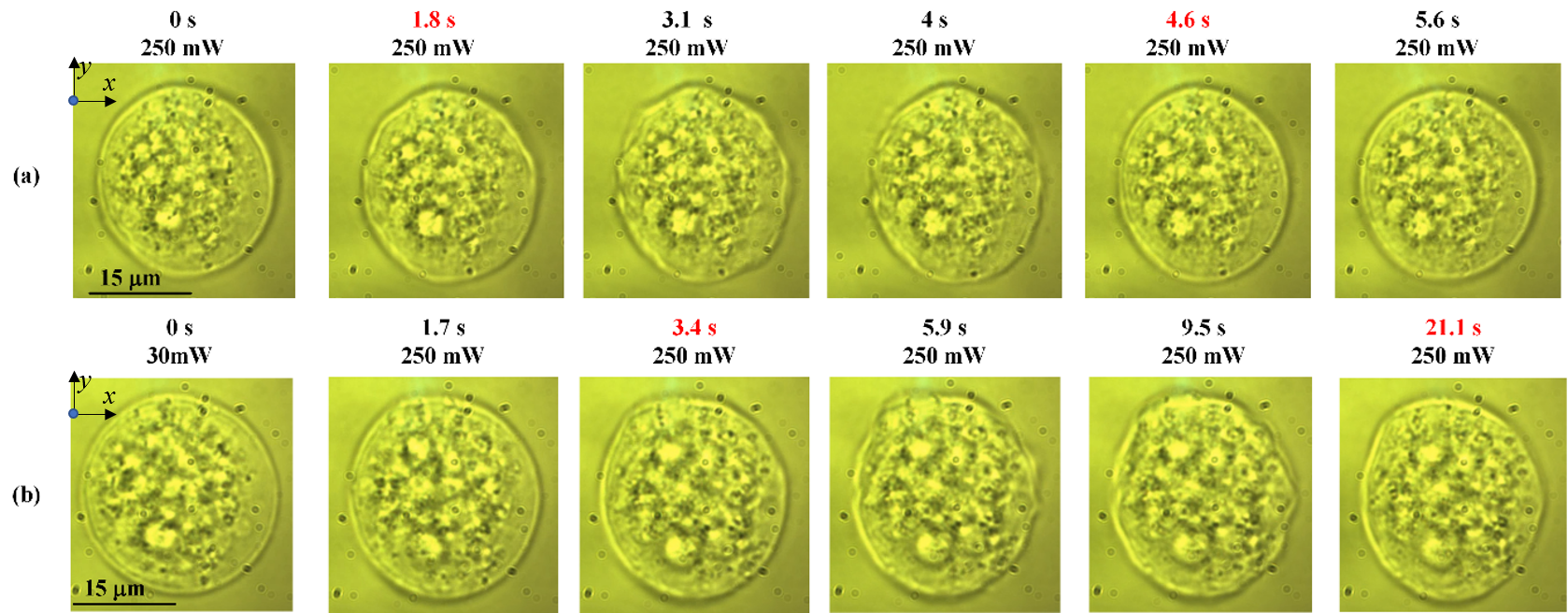


Fig S6 The MCF10A Pten-/-.cell membrane blebbing. When the MCF10A Pten-/-.cell was tested by the two cycles of optical force loading/unloading, as described in Fig. 2 in the main text, the membrane blebbing was observed when the power was raised to 250 mW in the second cycles (a). (b) The second time cell membrane blebbing of the same MCF10A Pten-/-.cell in (a). Each time the blebbing occurs, the optical power was fixed to be 250 mW until the cell recovered (at the end of each row of (a) and (b)).

1. **Observation of Cell photodamages: membrane blebbing (might be due to the trapping laser from AFMOTs) and rupture**

In the experiment, we do observe cell photodamages, which might be due to the light absorption or the intense optical forces applied to cells. It is well known that strong laser intensities can induce photodamages to cells[^4^](#_ENREF_4). Although the spot sizes (~10 μm) at the trap of AFMOTs are much larger than those (sub-micrometer) of traditional optical tweezers, resulting in a much lower intensity at the same power, we still observed photodamages in human breast cells, all at 250 mW. It is worthwhile to report these photodamages so that readers are aware of the limitations of the AFMOTs. The membrane blebbing were observed in 8 out of 13 MCF10A PTEN-/- cells and one out of 12 MCF10A cells, respectively. It was always observed at the maximum laser power of 250 mW, but not at lower powers. All of the cells recovered from the blebbing after 3~20 seconds with the laser power fixed at 250 mW. If the blebbing occurred to an MCF10A PTEN-/- cell during one loading/unloading cycle, there was a much higher likelihood for the blebbing to occur in the following cycle, and it also took longer for the blebbing to recover. A typical MCF10A PTEN-/- cell with membrane blebbing in two loading/unloading cycles are shown in Figure S6(a) and (b), respectively. For the MCF10A PTEN-/- cell in Figure S6, the membrane blebbing took ~3s and ~17s to recover in those two cycles, respectively. Since the cell membrane blebbing characterizes the execution phase of apoptosis[^5^](#_ENREF_5), it might indicate that trapping laser induces the apoptosis of MCF10A PTEN-/-. cells.


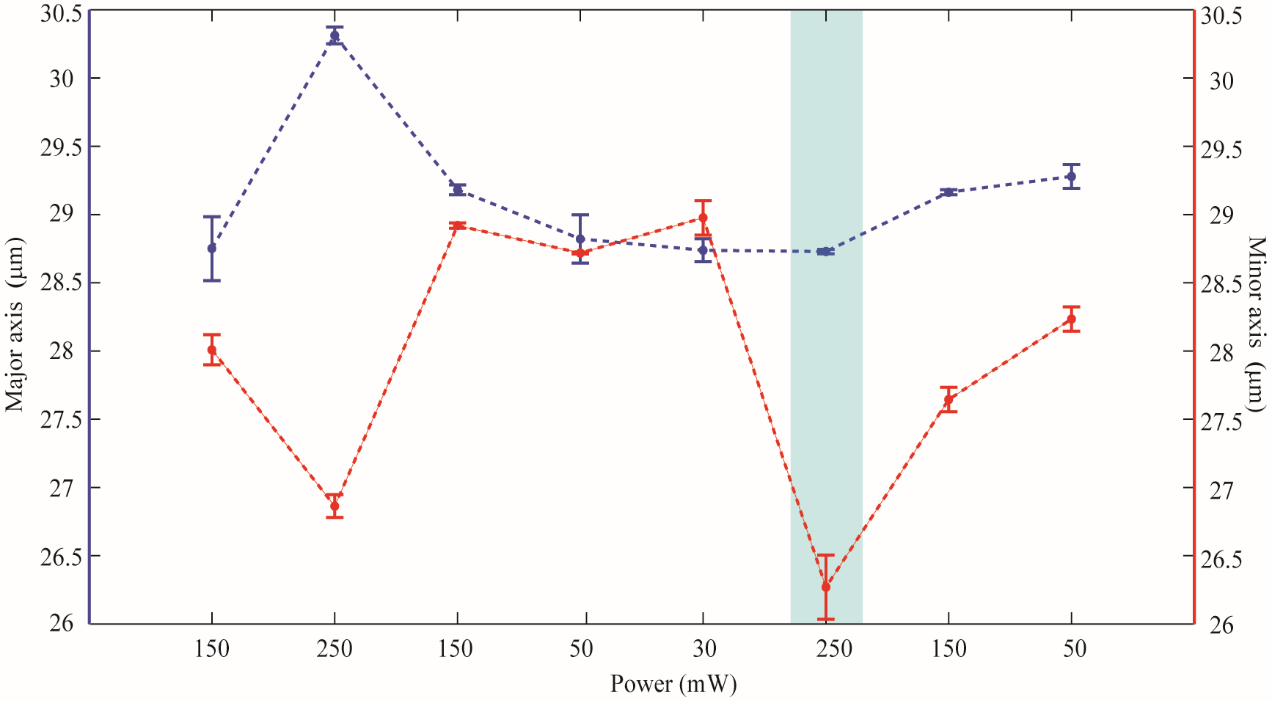


Fig S7 The major (dark blue) and minor (red) axis lengths of a MCF10A Pten-/-.cell [Figure S6(a)] measured in the two loading/unloading cycles. The shaded areas correspond to the measurements when cell membrane blebbing happens. This blebbing induced a large difference in the cell deformability between the first and second cycles.

It is noted that cell membrane blebbing could alter the deformability of MCF10A PTEN-/-. cells in AFMOTs. As shown in Figure S7, when the cell blebbing occurred, the major axis length of the MCF10A PTEN-/-. cell did not show any dependence on the optical power, although the deformation in the minor axis still depended on the optical power.


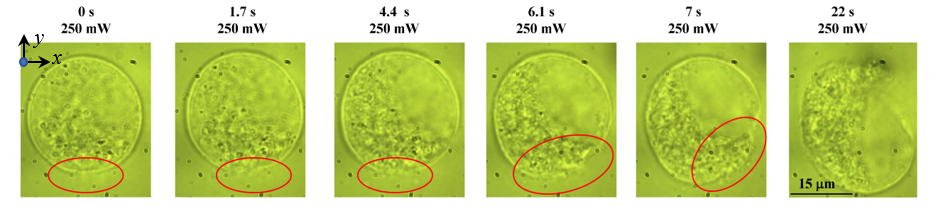


Fig S8 Cell rupture in AFMOTs at the optical power of 250 mW. Red circles indicate the location of the rupture.

Although it rarely happened, a high laser power could induce the cell membrane rupture. In all our experiments, we observed the membrane rupture of only 2 cells out of around 40 MCF10A and MCF10A PTEN-/- cells. Both were MCF10A PTEN-/- cells and the rupture occurred at the power of 250 mW, with one cell rupture process shown in Figure S8. The bottom area of the cell membrane was broken when the optical power was increased to 250 mW. The exact reason for cell membrane blebbing and rupture is still unknown and needs further investigation.

1. **References**

1 Ti, C., Ho-Thanh, M.-T., Wen, Q. & Liu, Y. Objective-lens-free Fiber-based Position Detection with Nanometer Resolution in a Fiber Optical Trapping System. *Scientific Reports* **7**, 13168, doi:10.1038/s41598-017-13205-6 (2017).

2 Liu, Y. & Yu, M. Investigation of inclined dual-fiber optical tweezers for 3D manipulation and force sensing. *Optics express* **17**, 13624-13638 (2009).

3 Liu, Y. & Yu, M. Multiple traps created with an inclined dual-fiber system. *Optics express* **17**, 21680-21690 (2009).

4 Neuman, K. C., Chadd, E. H., Liou, G. F., Bergman, K. & Block, S. M. Characterization of photodamage to Escherichia coli in optical traps. *Biophysical journal* **77**, 2856-2863 (1999).

5 Coleman, M. L. *et al.* Membrane blebbing during apoptosis results from caspase-mediated activation of ROCK I. *Nature cell biology* **3**, 339 (2001).
